# Antimicrobial Peptide LL-37 Facilitates Intracellular Uptake of RNA Aptamer Apt 21-2 Without Inducing an Inflammatory or Interferon Response

**DOI:** 10.3389/fimmu.2019.00857

**Published:** 2019-04-24

**Authors:** Tom Macleod, Joseph Ward, Adewonuola A. Alase, Charlie Bridgewood, Miriam Wittmann, Nicola J. Stonehouse

**Affiliations:** ^1^Institute of Molecular and Cellular Biology, Faculty of Biological Sciences, University of Leeds, Leeds, United Kingdom; ^2^Leeds Institute of Rheumatic and Musculoskeletal Medicine, Medicine and Health, University of Leeds, Leeds, United Kingdom; ^3^National Institute for Health Research, Leeds Biomedical Research Centre, Leeds Teaching Hospitals, Leeds, United Kingdom

**Keywords:** LL-37, RNA aptamer, skin, inflammation, interferon response, safety, complexes

## Abstract

RNA aptamers are synthetic single stranded RNA oligonucleotides that function analogously to antibodies. Recently, they have shown promise for use in treating inflammatory skin disease as, unlike antibody-based biologics, they are able to enter the skin following topical administration. However, it is important to understand the inflammatory milieu into which aptamers are delivered, as numerous immune-modulating mediators will be present at abnormal levels. LL-37 is an important immune-modifying protein upregulated in several inflammatory skin conditions, including psoriasis, rosacea and eczema. This inflammatory antimicrobial peptide is known to complex nucleic acids and induce both inflammatory and interferon responses from keratinocytes. Given the attractive notion of using RNA aptamers in topical medication and the prevalence of LL-37 in these inflammatory skin conditions, we examined the effect of LL-37 on the efficacy and safety of the anti-IL-17A RNA aptamer, Apt 21-2. LL-37 was demonstrated to complex with the RNA aptamer by electrophoretic mobility shift and filter binding assays. In contrast to free Apt 21-2, LL-37-complexed Apt 21-2 was observed to efficiently enter both keratinocytes and fibroblasts by confocal microscopy. Despite internalization of LL-37-complexed aptamers, measurement of inflammatory mediators and interferon stimulated genes showed LL-37-complexed Apt 21-2 remained immunologically inert in keratinocytes, fibroblasts, and peripheral blood mononuclear cells including infiltrating dendritic cells and monocytes. The findings of this study suggest RNA aptamers delivered into an inflammatory milieu rich in LL-37 may become complexed and subsequently internalized by surrounding cells in the skin. Whilst the results of this study indicate delivery of RNA aptamers into tissue rich in LL-37 should not cause an unwarranted inflammatory of interferon response, these results have significant implications for the efficacy of aptamers with regards to extracellular vs. intracellular targets that should be taken into consideration when developing treatment strategies utilizing RNA aptamers in inflamed tissue.

## Introduction

RNA aptamers are synthetic single stranded RNA oligonucleotides that bind targets with high specificity and affinity. Whilst they function like antibodies, there are several advantages presented by these molecules over their protein-based counterparts, boasting improved thermostability, reduced immunogenicity, and cheaper, more tractable production by chemical synthesis (1, 2). RNA aptamers are becoming an increasingly attractive immune-modulating tool for the treatment of disease. In particular, they have great potential for use in topical treatment of inflammatory skin conditions as they are small in size and therefore may effectively penetrate the skin, allowing direct treatment of diseased tissue without delivering a systemic dose of antibody-based biologics. This has been illustrated recently by delivering an anti-IL-23 RNA aptamer into epidermal compartments of porcine and *ex vivo* skin (3). However, when treating diseased tissue, it becomes important to consider the altered inflammatory milieu into which the drug is delivered.

In pathologically inflamed tissue, the upregulation of immune-modifying cytokines and proteins may impact on the efficacy of delivered RNA aptamers. One such protein is the anti-microbial peptide cathelicidin (LL-37) (4–8). LL-37 is derived from the precursor hCAP18, which is proteolysed to generate a biologically active C-terminal peptide of 37 amino acids, of which the first two are leucines (9). LL-37 is produced in the skin primarily by keratinocytes in response to invading micro-organisms and, once proteolytically activated, functions as a microbicidal peptide. This cationic peptide (with an α-helical structure) can bind the membranes of bacteria and enveloped viruses, polymerise on membrane surfaces and cause membrane disruption, killing invading organisms (10). In recent years, it has become evident LL-37 possesses numerous functions aside from its anti-microbial activity; many of which are immunomodulatory. Interestingly with regards to RNA aptamers, LL-37 has a high affinity for single and double stranded nucleic acids and is capable of enhancing inflammation through promoting toll-like receptor (TLR) activation (11–13). Furthermore, LL-37 has been shown to shuttle complexed nucleic acids across cell membranes (12, 14), primarily via receptor-mediated endocytosis. However, in keratinocytes, uptake seems to occur by mechanisms that do not require activation of specific receptors (15, 16), promoting inflammatory and interferon responses via both TLR and cytosolic nucleic acid sensors such as the cGAS-STING and RIG-I Like Receptor (RLR) pathways (17, 18).

LL-37 is found over-expressed in many of the most common inflammatory skin conditions, including psoriasis, rosacea, and eczema (5, 6, 19). These conditions together account for a significant percentage of patients treated for skin-related illnesses, with ~3%, 10–20%, and 10% of the population suffering from psoriasis, rosacea and eczema, respectively. Recently, the advent of biologic medicine has facilitated highly effective treatment strategies for these conditions, yet the expense and difficulties found in effective delivery limits biologic treatment to the most severe of cases. Topically applied aptamer-based treatments provide a cheaper and arguably more effective alternative to protein-based biologics that would open the field of biologic medicine to a much larger percentage of patients. IL-17A is a pro-inflammatory protein that plays a central role in initiating and perpetuating inflammation in psoriasis, and has been targeted with great success using antibody-based biologic treatments (20–22). Expressed by infiltrating immune cells in the skin, IL-17 cytokines act on surrounding keratinocytes and fibroblasts to induce expression of angiogenic and inflammatory mediators crucial in the development of psoriatic lesions (23). The anti-IL-17A RNA aptamer Apt 21-2 has also been shown to effectively bind IL-17A, and we and others have previously illustrated that Apt 21-2 may be suitable for use in treating psoriatic plaques (24, 25). Given the attractive notion of using RNA aptamers in topical medication and the prevalence of LL-37 in these inflammatory skin conditions, it is of great importance to examine the effect of LL-37 on RNA aptamer efficacy and safety. This work investigates the interaction between Apt 21-2 and LL-37, and the consequent effects on aptamer uptake and immune activation.

## Methods

### Reagents

RNA Aptamer Apt 21-2 (25) was synthesized to order by Dharmacon GE Healthcare as 33 nucleotides (5′ GGUCUAGCCGGAGGAGUCAGUAAUCGGUAGACC 3′) with 2′ fluoro-modified cytosine and uracil. A fluorescently tagged Apt 21-2 was also synthesized by addition of a single Cy3 molecule on the 5′ end of the aptamer (Apt 21-2 Cy3) (24). Fluorochrome-conjugated antibodies were obtained from Miltenyi Biotech (HLA-DR-FITC, CD11c-VioBlue, CD14-VioBlue, CD19-VioBlue, IFNα-APC) or BioLegend (CD303-PE-Cy7, CD123-BV711). For analysis of intracellular cytokines by flow cytometry, cytokine secretion was inhibited by GolgiPlug (BD Biosciences).

### Primary Cell Isolation, Cell Culture, and Ethics

Primary keratinocytes and fibroblasts from healthy donors were purchased from PromoCell or isolated from healthy volunteers respectively and were cultured as described previously (26). The participants' samples used for this study were collected under ethical approval, REC 10/H1306/88, National Research Ethics Committee Yorkshire and Humber-Leeds East. All experiments were performed in accordance with relevant guidelines and regulations.

### PBMC Isolation

Whole blood anti-coagulated in heparin was collected from healthy volunteers and PBMCs were isolated by density gradient, followed by centrifugation using Greiner Leuco-Sep tubes (Sigma Aldrich, Gillingham, UK). Isolated PBMCs were washed in MACS buffer (D-PBS, 2 mM EDTA, 0.5% BSA) followed by 1x wash in PBS. Cells were resuspended in RPMI 1640 (10% FCS, 1% penicillin/streptomycin), plated in 24 well plates, and immediately stimulated.

### Flow Cytometry

PBMCs were stimulated as detailed in the results in the presence of GolgiPlug. Following stimulation, cells were washed in PBS and resuspended in blocking buffer (10% mouse serum and 1% IgG) for 15 min at 4°C. Cells were then stained for surface antigens (HLA-DR, CD11c, CD14, CD19, CD303, CD123) for 30 min at 4°C. Cells were washed and fixed and permeabilized using IntraPrep kit (Beckman Coulter) according to the manufacturer's instructions. Cells were then washed and stained for intracellular IFNα for 30 min at 4°C. Finally, cells were washed, resuspended in PBS and analyzed by a BD LRSII flow cytometer (BD Biosciences). Plasmacytoid dendritic cells (pDCs) were identified as a HLA-DR^high^, CD11c^low^, CD14^low^, CD19^low^, CD303^high^, CD123^high^ population (gating strategy outlined in Figure S1).

### Confocal Microscopy

Cells were grown on poly-D-lycine coated coverslips to the desired confluency prior to stimulation as detailed in the results. Following stimulation, cells were subject to an acid wash (200 mM acetic acid, 150 mM NaCl), were fixed in 4% paraformaldehyde, and permeabilized with 0.3% saponin BSA PBST before mounting on glass slides in mountant containing DAPI. Cells were then imaged with a LSM880 confocal microscope. Images were processed in Zen software.

### Quantitative PCR

Quantitative real-time PCR was carried out by a QuantStudio 5 Real Time PCR System (ThermoFischer) and a ΔΔCT-analysis formed from the generation of standard curves for the housekeeping genes and genes of interest. RNA isolation was carried out using the Direct-zol RNA MiniPrep kit (Zymo Research). cDNA was generated by SuperScript II reverse transcriptase (Thermo Fischer Scientific) according to the manufacturer's protocol. Qiagen QuantiFast SYBR green PCR was used to carry out the qRT-PCR according to the manufacturer's protocol.

### ELISA

Nunc-ImmunoTM MicroWellTM 96 well plates (SIGMA) were coated with capture antibody and ELISA proceeded as detailed in manufacturer instructions using IL-8 ELISA MAX Standard ELISA kit (BioLegend, Hatfield, UK), IFNα ELISA using MT1/3/5 capture antibody and MT2/4/6 detection antibody (Mabtech), and CXCL10 ELISA DuoSet (R&D Systems).

### 5′ ^32^P Labeling of Apt 21-2

Unlabeled Apt 21-2 (1 μg) synthesized by Dharmacon was incubated at 37°C for 30 min in a reaction containing 2 μl of T4 Poly nucleotide kinase (PNK) (NEB), 2 μl PNK buffer and ~30 μCi of ^32^p UTP in a total reaction size of 20 μl. Following incubation, the reaction was terminated by heating to 65°C for 10 min and purified by ethanol precipitation and resuspension in nuclease free water.

### Electrophoretic Mobility Shift Assay

^32^P labeled aptamer and LL-37 were mixed at the concentrations stated and incubated for 1 h on ice. Native polyacrylamide gel (7%) was prepared in TBE buffer and electrophoresis was carried out in TBE buffer. Samples were loaded with 60 mM KCl, 10 mM TRIS (pH 7.6), 40% glycerol and 0.01% bromophenol blue. Following completion of the separation, the gels were fixed in 12% methanol and 10% acetic acid in dH_2_O, before drying in a vacuum pumped gel dryer (Biorad) and imaged by exposure to a phosphoscreen.

### Filter Binding Assay

^32^P labeled aptamer and LL-37 were mixed at the concentrations stated and incubated for 1 h on ice. Samples were drawn through stacked nitrocellulose and nylon membranes using a slot-blot device (Biorad). Filters were dried and imaged by exposure to a phosphoscreen.

### RNA Urea Gels

Urea gels (7% polyacrylamide, 7 M urea) were prepared in TBE buffer for analysis of RNA stability. Samples were prepared by addition of equal volume 2x Novex^®^ TBE-Urea Sample Buffer (ThermoFischer), incubated at 85°C, then cooled on ice prior to loading on the urea gels. Gels were stained with 0.2% methylene blue (0.4 M sodium acetate, 0.4 M acetic acid) for visualization of RNA.

### Statistical Analysis

Statistical analysis was performed using GraphPad Prism 7 software. Data were analyzed by one-way ANOVA to determine overall differences, and a Tukey *post hoc* test to determine statistical significance between groups.

## Results

### LL-37 Interacts With RNA Aptamer Apt 21-2

**Figure 1:**
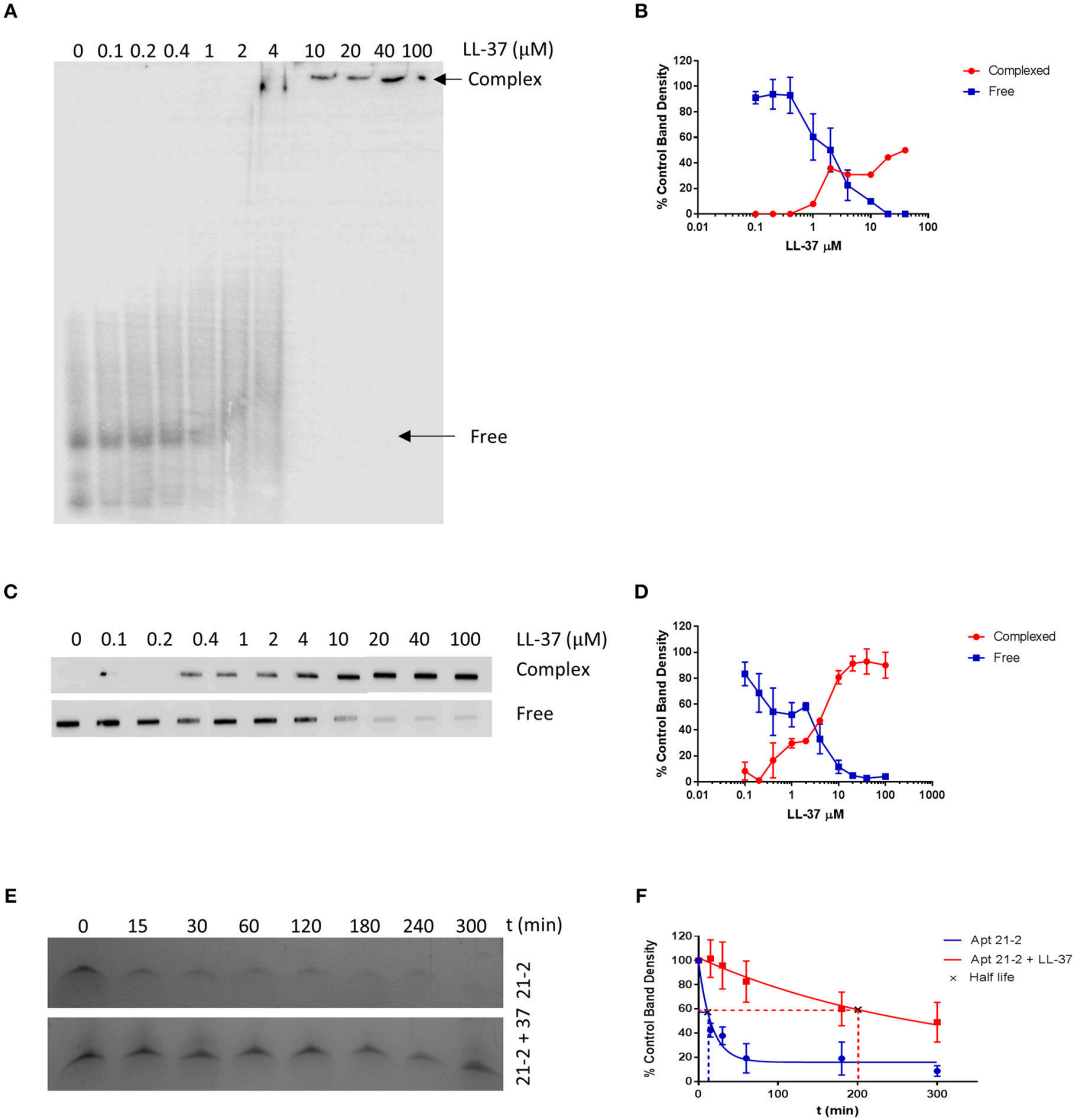
Samples of Apt 21-2 (100 nM) combined with LL-37 (0 to 100 μM as indicated above lanes) were incubated for 1 h on ice. Samples were then separated on a 7% native polyacrylamide gel **(A)** or by filter binding assay **(C)**. Images are representative of 2 independent experiments. Densitometry was measured by ImageJ software and plotted in GraphPad Prism as percentage density of control RNA band with no added LL-37 to estimate K_D._ Data shown are mean ± SD (*n* = 2) **(B,D)**. Apt 21-2 (1 μg) incubated with fetal calf serum at 37°C in the presence or absence of LL-37 (10 μg) for 5 h. Samples taken at indicated time points, separated on a 7% polyacrylamide urea gel and visualized using methylene blue stain. Image representative of 3 independent experiments **(E)**. Densitometry calculated by ImageJ software and normalized to t = 0 band density as control. Band density plotted and half-life calculated in GraphPad Prism. Data shown are mean ± SD (*n* = 3) **(F)**.

LL-37 has been shown to interact with nucleic acids including single and double stranded RNA, and this interaction is thought to be mediated via positively charged residues on LL-37 (11, 13, 27). It therefore seems plausible for LL-37 to interact and complex with RNA aptamers. To explore whether this is the case, we incubated Apt 21-2 5' end labeled with ^32^P UTP (100 nM) with increasing concentrations of LL-37 and separated samples by electrophoretic mobility shift assay (EMSA). A large observable shift in ^32^P-labeled aptamer occurs as the concentration of LL-37 is increased, indicative of a higher order protein and aptamer complex (Figure 1A). This interaction was also observed when conducting a filter binding assay with the same samples (Figure 1C). There is evidence of protein:RNA complex formation at approximately the same concentration as the observed shift in EMSA. Densitometry analysis of band density in both EMSA and the filter binding assay show a 50% reduction in free aptamer at ~2 μM, indicating an interaction between Apt 21-2 and LL-37 with an apparent K_D_ of 2 μM (Figures 1B,D).

LL-37-complexed RNA has been reported in the literature to be less susceptible to degradation. We questioned whether this would also be true of LL-37-complexed Apt 21-2. We therefore incubated Apt 21-2 in the presence or absence of LL-37 in fetal calf serum for 5 h at 37°C and analyzed aptamer degradation by separation of the samples on denaturing (urea) gels. Within the 1st h Apt 21-2 alone had significantly degraded. However, addition of LL-37 reduced the extent of degradation (Figure 1E). Indeed, half-life of aptamer alone was calculated as 11.5 ± 5.65 min, whilst LL-37-complexed aptamer was calculated to have a half-life of 202.4 ± 82.95 min (Figure 1F).

### LL-37 Facilitates Internalization of Apt 21-2 in Keratinocytes and Fibroblasts

LL-37 is known to cross plasma membranes through a variety of mechanisms, and in doing so can facilitate internalization of its binding partners. This has been shown to occur with poly(I:C) in keratinocytes and both viral dsRNA and self-RNA released from dying cells, as well as with other non-nucleic acid binding partners such as LPS (28). Once LL-37 was identified to associate with Apt 21-2, we next examined whether this interaction (in the presence of primary keratinocytes and fibroblasts) might promote internalization of the RNA aptamer.

**Figure 2:**
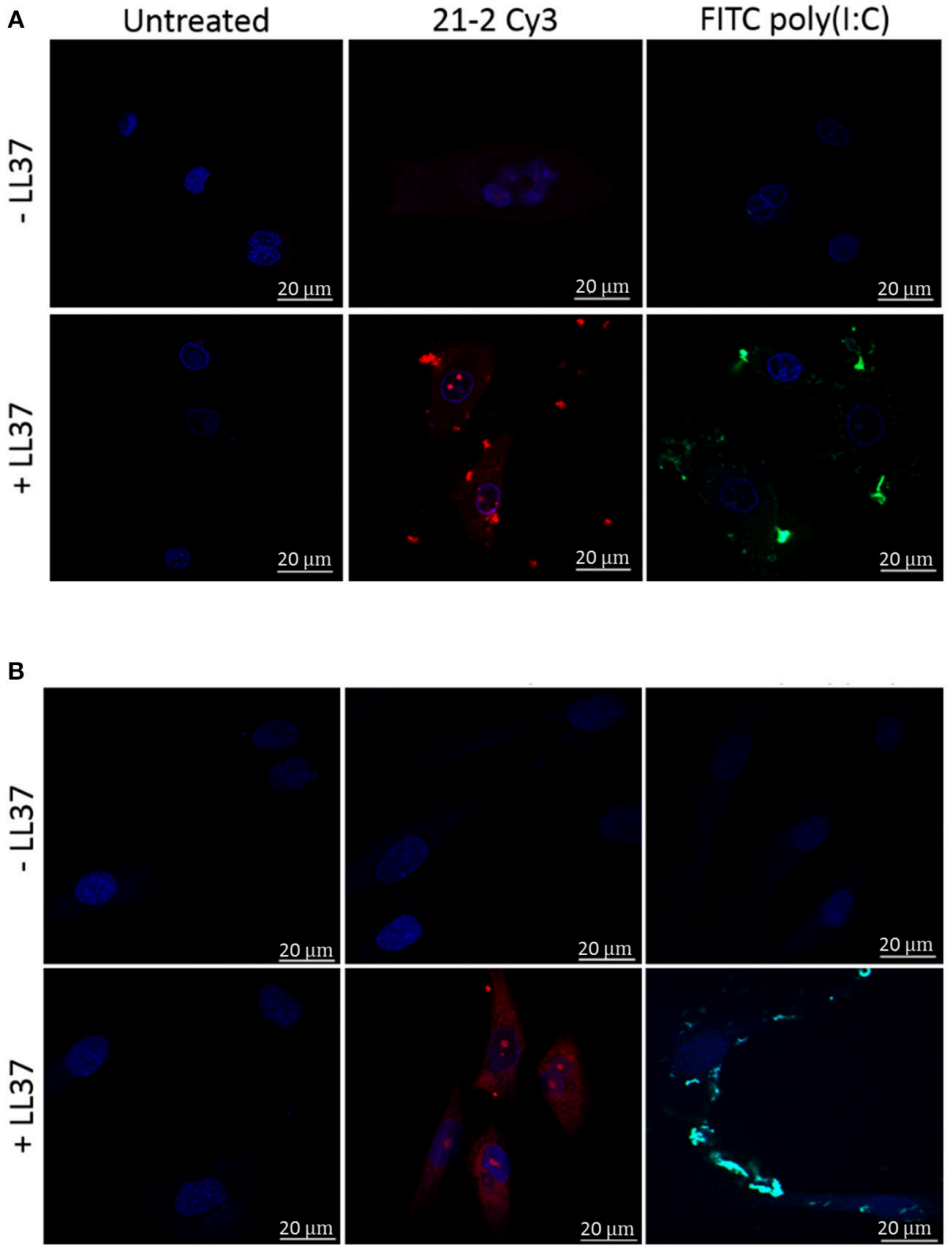
Healthy primary keratinocytes **(A)** and fibroblasts **(B)** were treated with Apt-21-2 Cy3 (100 nM; red) or FITC-conjugated poly(I:C) (1 μg/ml; green) in the presence or absence of LL-37 (2.5 μM) for 24 h. Cells were washed with acid to remove extracellular RNA and imaged by confocal microscopy to assess uptake of Apt 21-2 and poly(I:C). Nuclei were visualized with 4′-6-diamidion-2-phenylidole (DAPI) (bars = 20 μm). Images are representative of three independent experiments.

Primary keratinocytes and fibroblasts were treated with Cy3-labeled Apt 21-2 (Apt 21-2 Cy3) and FITC-labeled poly(I:C) in the presence or absence of LL-37 before analysis by confocal microscopy. As previously reported, evidence of uptake (to a low level) by keratinocytes was observable when Apt 21-2 Cy3 was added to cells alone (Figure 2A). Conversely, no uptake was observed by the fibroblasts (Figure 2B). However, with the addition of 2.5 μM LL-37 internalization of Apt 21-2 Cy3 was greatly enhanced in both keratinocytes and fibroblasts, with evidence of punctate aggregation and diffuse cytosolic staining closely resembling that observed upon addition of both LL-37 and FITC-poly(I:C). Indeed, whilst uptake of aptamer alone was not observed in fibroblasts, significant internalization was observed when added with LL-37 (Figure 2).

**Figure 3:**
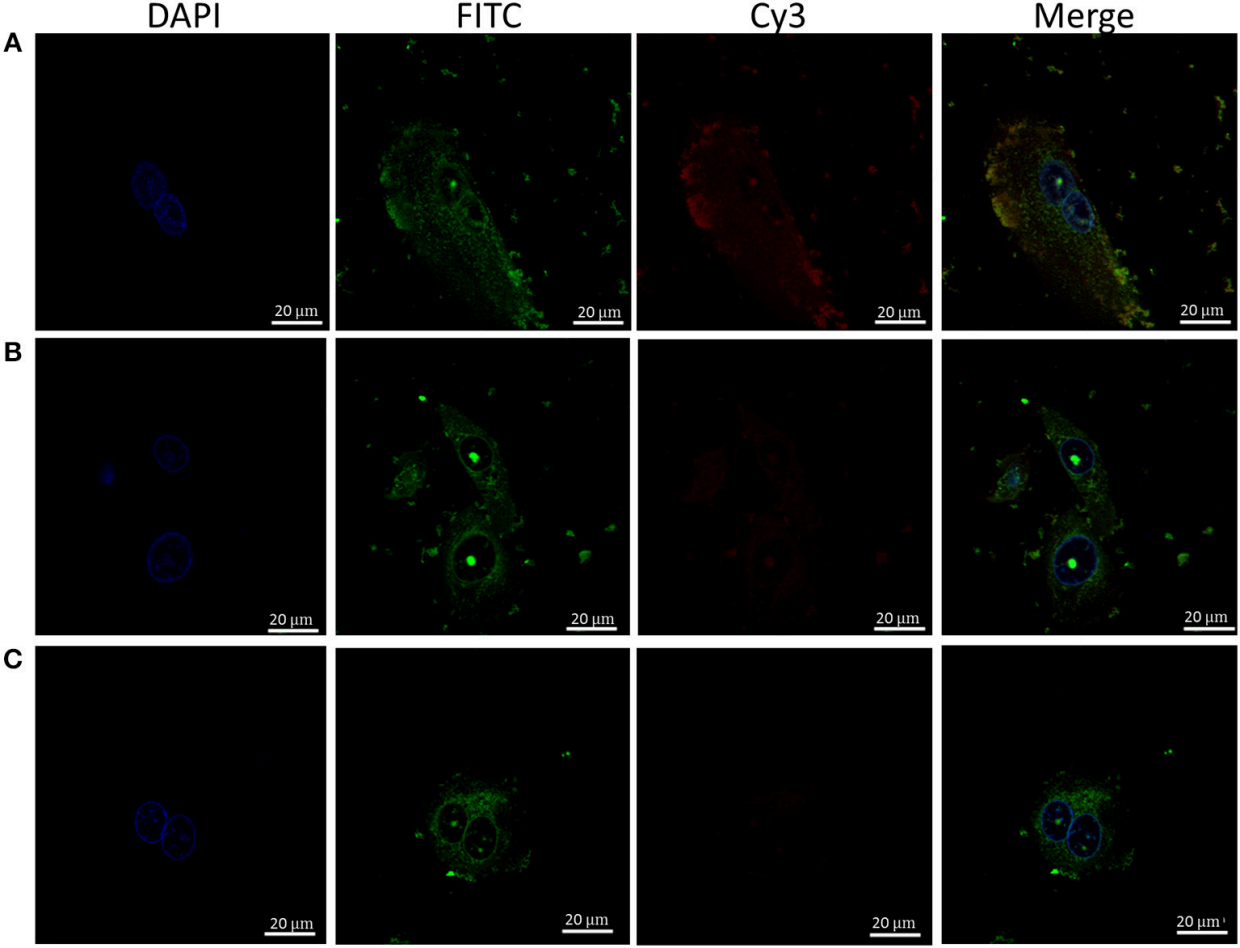
Healthy primary keratinocytes were treated with Apt-21-2 Cy3 (100 nM; red) and FITC-labeled LL-37 (2.5 μM; green) with either 0 μM **(A)**, 1 μM **(B)**, or 5 μM **(C)** unlabeled Apt 21-2 to compete with Apt 21-2 Cy3 for binding with LL-37. Cells were washed with acid to remove extracellular RNA and imaged by confocal microscopy to assess uptake of aptamer. Nuclei were visualized with DAPI (bars = 20 μm). Images are representative of 2 independent experiments.

Given the hydrophobic nature of cyanine dyes and the propensity of LL-37 to interact with various molecular partners, it is important to confirm the observed interaction between LL-37 and Apt 21-2 Cy3 is due to interaction with the aptamer RNA rather than the cyanine label. To this end, reactions of FITC-LL-37 and Apt 21-2 Cy3 were spiked with increasing concentrations of unlabeled Apt 21-2 to compete for binding with LL-37 before addition to cells (Figures 3B,C). As shown in Figure 3A, Apt 21-2 Cy3 appears to co-localize with the aggregated FITC-LL-37, both in extracellular and intracellular aggregates. However, a decrease in Cy3 fluorescence was observed corresponding with increase in concentration of unlabeled Apt 21-2, suggesting the uptake observed in Figures 2A,B is due to interaction between LL-37 and RNA rather than LL-37 and Cy3. Furthermore, addition of LL-37-FITC suggests co-localization of LL-37 and Apt 21-2 (Figure 3A), corroborating the results observed by EMSA and the filter binding assay (Figure 1).

### Apt 21-2 Does Not Induce an Inflammatory or Interferon Response When Combined With LL-37

Once associated with LL-37, dsRNA/LL-37 complexes can facilitate an inflammatory and interferon response by enhancing activation of TLRs after internalization of complexed RNA. This has been illustrated for co-stimulation of LL-37 with poly(I:C), self-RNA and DNA (29). Although small RNA aptamers are known to be immunologically inert, the possibility of their interaction with LL-37 allowing an immunological response must be considered. We therefore stimulated primary keratinocytes and fibroblasts with either Apt 21-2 or poly(I:C) in the presence or absence of LL-37 and measured both IL-8 secretion and mRNA expression of skin-relevant interferon stimulated genes (ISGs) MxA, CXCL10, GBP-1, and the tissue-derived IFNλ (in keratinocytes), which has a significant role in tissue-based antiviral activity (30, 31).

**Figure 4:**
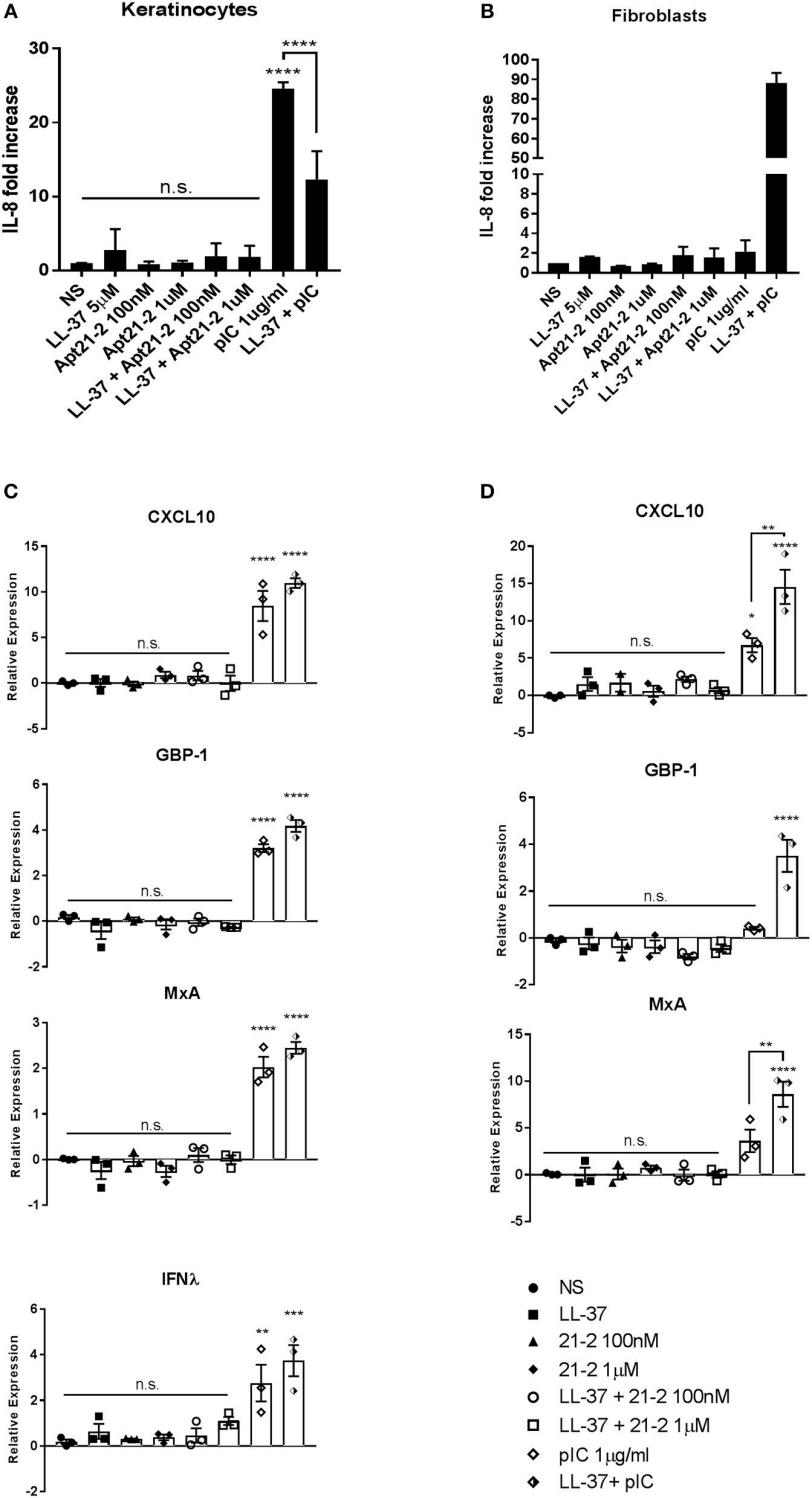
Healthy primary keratinocytes **(A,C)** and fibroblasts **(B,D)** were grown to 80% confluence in 24 well plates and treated with LL-37 (5 μM), Apt 21-2 (100 nM, 1 μM), LL-37 + Apt 21-2 (5 μM + 100 nM and 1 μM, respectively), poly(I:C) (1 μg/ml), poly(I:C) + LL-37 (1 μg/ml + 5 μM), or left untreated (NS). To assess inflammatory response supernatants were harvested 24 h post-stimulation and IL-8 measured by ELISA **(A,B)**. Data shown are mean ± SD from independent experiments. *n* = 3, ANOVA, ^****^p < 0.0001 **(A)** *n* = 2 **(B)**. To assess interferon response RNA was harvested at 6 h post stimulation and mRNA expression of the interferon-stimulated genes CXCL10, GBP-1, MxA, and IFNλ was measured by qPCR normalized to U6 housekeeping gene presented as ΔΔCq. ANOVA, ^*^p < 0.05, ^**^p < 0.01, ^***^p < 0.001, ^****^p < 0.0001. Data shown are mean ± SEM with individual data points of independent experiments, *n* = 3 **(C,D)**.

As has been previously reported, treatment with poly(I:C) alone induced a strong response from primary keratinocytes, eliciting both IL-8 secretion and ISG expression (Figures 4A,C). Whilst addition of LL-37 alone had little effect on either IL-8 secretion or ISG expression, an additive effect was observed in ISG expression when added in combination with poly(I:C) to keratinocytes, and a synergistic increase in both ISG expression and IL-8 secretion by fibroblasts. Primary keratinocytes, however, appeared to secrete less IL-8 when challenged with both LL-37 and poly(I:C) (Figure 4). Whilst this does not seem to fit the trend of our other results, this inhibition of poly(I:C)-induced IL-8 secretion by LL-37 in keratinocytes has been previously reported in the literature (13). Contrary to poly(I:C), stimulation with Apt 21-2 did not induce IL-8 secretion or up-regulation of measured ISGs. Furthermore, the addition of LL-37 and Apt 21-2 in combination had no significant effect on IL-8 secretion or ISG expression in either keratinocytes or fibroblasts (Figure 4).

**Figure 5:**
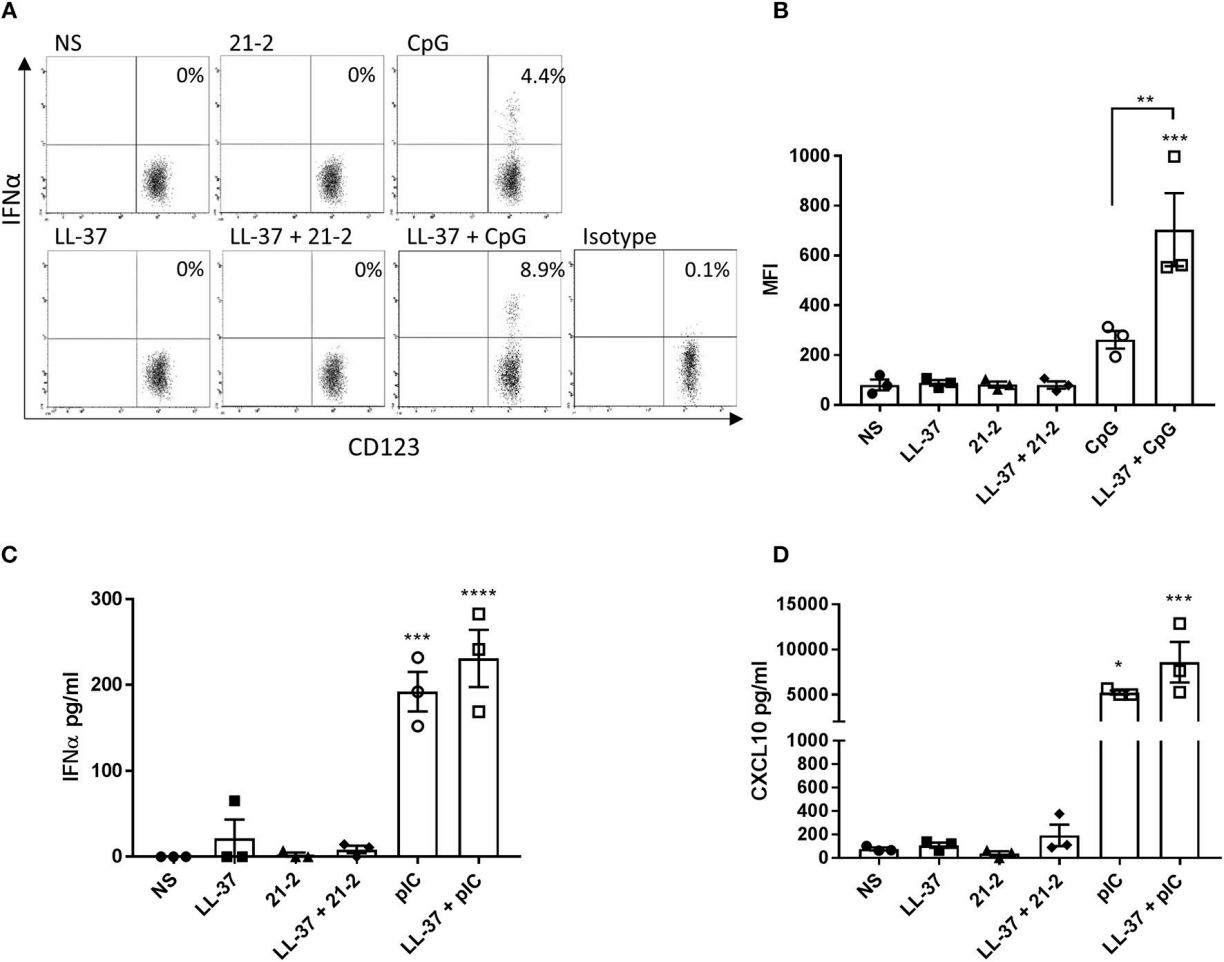
PBMCs were isolated from whole blood and treated with LL-37 (2.5 μM), Apt 21-2 (100 nM; 21-2), CpG ODN (2.5 μM; CpG), LL-37 + Apt 21-2 (2.5 μM + 100 nM), LL-37 + CpG ODN (2.5 μM each), or left untreated (NS) for 12 h at 37°C 5% CO_2_. The isotype control was treated with CpG (2.5 μM). After 1 h of stimulation, GolgiPlug was added to all cells (1 μl per ml of media). Following stimulation, the percentage of IFNα^+^ pDCs was determined by flow cytometry. pDCs were identified using the gating strategy outlined in Figure S1. A representative set of dot plots for one donor is shown **(A)** with a graph plotting mean fluorescence intensity (MFI) of IFNα for each donor **(B)**. Data shown are mean ± SEM with individual data points of independent donors, *n* = 3, ANOVA, ^**^p < 0.01, ^***^p < 0.001. **C-** PBMCs were stimulated as in **A** but substituting CpG ODN with poly(I:C) (100 μg/ml) and without addition of GolgiPlug. Cells were incubated for 24 h at 37°C 5% CO_2_. Supernatants were harvested and tested for IFNα **(C)** and CXCL10 **(D)** by ELISA. Data shown are mean ± SEM with individual points of independent donors. *n* = 3 ^*^p < 0.05, ^***^p < 0.001, ^****^p < 0.0001.

In addition to keratinocytes and fibroblasts, immune cells also infiltrate into the dermis and epidermis, and are often found in increased numbers during inflammation. Of these, plasmacytoid dendritic cells (pDCs) and monocytes/macrophages are known as significant producers of type 1 interferon and are known to respond to LL-37-complexed nucleic acids (7, 11, 18, 27). We therefore examined the response of pDCs to LL-37-complexed Apt 21-2. Human PBMCs were stimulated with either Apt 21-2 or the TLR9 agonist CpG oligodeoxynucleotide (ODN) in the presence or absence of LL-37 for 12 h, and pDC intracellular IFNα production was assessed by flow cytometry. Additionally, PBMCs were treated with either Apt 21-2 or the TLR3 agonist poly(I:C) in the presence or absence of LL-37 for 24 h, and secreted IFNα and the interferon stimulated chemokine CXCL10 were measured by ELISA (Figures 5C,D). As shown in Figure 5A, whilst stimulation with CpG ODN induced a modest increase in the percentage of IFNα^+^ pDCs, no response was measured following Apt 21-2 stimulation. When stimulated with both LL-37 and CpG ODN, a significant increase in the percentage of IFNα^+^ pDCs was observed over CpG ODN stimulation alone, as has been previously reported (7) (Figure 5B). In contrast, no IFNα^+^ pDCs were identifiable following stimulation with LL-37 and Apt 21-2 in combination (Figure 5A). In agreement with these findings, PBMCs treated with poly(I:C) secreted significant amounts of both IFNα and CXCL10, and these levels increased when treated in combination with LL-37. However, treatment with Apt 21-2 did not cause an elevation in secretion of IFNα or CXCL10 above that of non-stimulated cells, and the addition of LL-37 in combination with Apt 21-2 had no significant effect on the secretion of either IFNα or CXCL10 (Figures 5C,D). These results suggest that despite the interaction between Apt 21-2 and LL-37 and the increase in internalization of the complexes, Apt 21-2 remains immunologically inert when present with LL-37, unable to elicit an interferon or inflammatory response. This remains true for both skin resident and infiltrating immune cells.

## Discussion

In this study, we initially sought to determine whether LL-37, a pro-inflammatory protein well documented to interact with nucleic acid, can also complex with a single stranded RNA aptamer. We tested this by electrophoretic mobility shift assay, filter binding assay, and confocal microscopy, all of which provided evidence of interaction. Analysis by EMSA and filter binding established that, in a controlled reaction, LL-37 interacts with Apt 21-2 with an apparent K_D_ of ~2 μM, and when added to cells in combination we observed strong co-localization of the aptamer and protein. This observation is perhaps not surprising as LL-37 is well documented to complex with both single and double stranded self-RNA and exogenous RNA, in addition to DNA (11, 13, 27). Indeed, Ganguly et al. postulated that LL-37 may preferentially bind structured RNA containing double stranded regions and stem loops. However, by illustrating the ability of LL-37 to complex small chemically modified RNA aptamers we bring to light the possibility that any RNA aptamers delivered into an environment rich in LL-37 may become complexed and potentially sequestered by the antimicrobial peptide. Keratinocytes have been shown to significantly increase production of LL-37 in response to cytokines associated with psoriasis (32). Whilst exact concentrations of LL-37 in the skin is unclear, it has been observed that psoriatic lesions contain a median of 304 μM LL-37 (5). Delivering an aptamer into such high concentrations, in this case ~150 times higher than the apparent K_D_, it seems likely that a proportion of the delivered aptamer will become complexed. Whilst this study was conducted in the context of skin-based inflammation, LL-37 expression is found over-expressed in several diseases and tissues, including inflammatory bowel disease and rheumatoid arthritis, and has been measured up to ~6 μM in bronchoalveolar lavage fluid extracted from infants suffering systemic inflammation, and so should be considered when treating any inflamed area (33–35). Even in healthy human sweat, LL-37 can be found at a concentration of ~1 μM, which may have considerable implications when using a topically administered RNA aptamer (36).

A significant finding of the work conducted in this study is the observation that LL-37-complexed aptamer is efficiently internalized by both keratinocytes and fibroblasts. Whilst keratinocytes are known to actively take up extracellular components quite readily by macropinocytosis, fibroblasts are not known to do so. Indeed, our previous work has demonstrated that when added to keratinocytes and fibroblasts alone, keratinocytes take up RNA aptamers, but the fibroblasts do not (24). However, as demonstrated here, when complexed with LL-37, the aptamer is internalized by both fibroblasts and keratinocytes, with confocal microscopy exhibiting striking intracellular staining in both keratinocytes and fibroblasts with a slight punctate appearance in keratinocytes. Internalization of LL-37-complexed nucleic acid has been previously reported in the context of keratinocytes and dendritic cells, however, to our knowledge this is the first time it has been described in fibroblasts (11, 17, 27). Indeed, these novel observations have significant implications when considered in the context of using RNA aptamers to treat inflammatory skin conditions and may influence how and where RNA aptamers might be delivered for treatment of extracellular or intracellular targets. As the results presented in this work show both keratinocytes and fibroblasts will internalize RNA aptamers complexed by LL-37, an extracellular target in either the dermis or epidermis may prove difficult to treat in this manner in inflamed skin tissue. Conversely, as internalization appears to be so effective in the presence of LL-37, it may be possible to utilize this mechanism as a method of targeting intracellular pathways and molecules. Considering these observations, it seems natural to suggest when using RNA aptamers to treat inflammatory skin conditions where LL-37 is strongly expressed that intracellular targets may be more desirable than extracellular. We have previously reported that free RNA aptamers taken up by cells enter the endosomal/lysosomal pathway, however whether these aptamers escaped the endosomal network is unclear (37). It is also unclear as to the effect that LL-37 complexing might have on intracellular trafficking of internalized complexes, and whether complexed aptamers might be able to access cytosolic targets. These possibilities should be further explored by delineating the mechanism by which LL-37 facilitates entry of complexed aptamers to facilitate identification of the fate of internalized complexes.

LL-37 has been reported to increase stability of complexed RNA, inhibiting RNase-mediated degradation (27). By incubating LL-37-complexed and free Apt 21-2 with fetal calf serum we have illustrated this is also true for LL-37-complexed RNA aptamers. This may have implications for the efficacy of RNA aptamers in inflammatory milieu as they may persist for longer in a LL-37 rich environment if provided protection by complexing. Indeed, this may prove beneficial if complexed aptamers remain functional, however, this is currently unknown. As LL-37-nucleic acid complexes have been observed to dissociate once internalized into acidic endosomes, it seems plausible that complexed and internalized aptamers may also be released and so available to bind targets (38). It may, therefore, be interesting to examine the kinetics of binding between LL-37 and RNA aptamers under various physiological conditions as this may provide key information on the availability of RNA aptamers when present in LL-37-rich tissue.

An important consideration which comes to light from demonstrating that RNA aptamers are both complexed and internalized with LL-37 is the effect this has on immune activation. RNA aptamers are considered immunologically inert, however, with LL-37 known to be an immunomodulatory protein that can significantly enhance the inflammatory properties of nucleic acids through mechanisms that are not entirely characterized, it is important to explore whether LL-37-complexed RNA aptamers become immunologically stimulatory. Previous work has shown that LL-37 complexed with ssRNA can initiate inflammatory signaling through TLR7 and TLR8 in pDCs once delivered into endosomal compartments, however, the results obtained in our study show that whilst LL-37-complexed aptamers were delivered intracellularly, no activation of cells was observed following treatment of either healthy pDCs, PBMCs, keratinocytes or fibroblasts with complexed aptamer (27). Whilst expression of TLR7 and TLR8 in healthy keratinocytes is not clearly defined, with contradictory evidence published in the literature, fibroblasts reportedly express both, and the pDC response to LL-37-complexed ssRNA has been previously described (27, 39). In addition to pDCs, monocytes are also known to infiltrate into the epidermis in inflamed tissue and can also contribute to IFN production in response to LL-37-complexed nucleic acids (18). Whilst pDCs effectively respond to ssRNA, they are poor expressers of TLR3 and therefore do not respond well to dsRNA (40). Monocytes/macrophages, however, express high levels of TLR3 and generate IFN in response to dsRNA (41). Despite this, neither pDCs nor isolated PBMCs (containing pDCs, conventional DCs and monocytes/macrophages) generated an IFN response to LL-37-complexed aptamer. These results therefore suggest aptamer 21-2:LL-37 complexes are unable to activate TLR7/8 or TLR3. LL-37 is thought to enhance activation of TLR3 through complexing dsRNA and producing crystalline structures that more effectively initiate TLR3 by engaging several receptors, inducing receptor clustering and immune amplification (42). The efficacy of these crystalline structures was found to depend on the length of dsRNA present in the crystals, so it is possible that Apt 21-2 does not contain long enough dsRNA tracts to form the correct crystal structure with LL-37 and so does not activate TLR3 in this manner. However, larger RNA aptamers may contain longer stretches of duplexed RNA, therefore further research to examine the effect of aptamer length on TLR activation may be necessary.

In conclusion, this work has illustrated the importance of understanding the environment into which an RNA aptamer is being delivered when treating inflammatory disease. In particular, it has shown that RNA aptamers delivered into inflamed tissue rich in the anti-microbial peptide LL-37 will become complexed and internalized by surrounding cells. Despite evidence of complexing and internalization, we did not observe any inflammatory or interferon response from keratinocytes, fibroblasts, or PBMCs, suggesting RNA aptamers should be safe for use when delivered into inflamed skin. However, the observation that LL-37-complexed aptamers are internalized by surrounding cells should be taken into consideration when developing an RNA aptamer-based treatment for an extracellular target in inflamed tissue with high levels of LL-37, as cells may sequester complexed aptamers away from their targets.

## Author Contributions

TM, JW, CB, and AA undertook experimental work. NS and MW designed and supervised the study. All authors contributed to writing the manuscript, with TM taking the leading role.

### Conflict of Interest Statement

The authors declare that the research was conducted in the absence of any commercial or financial relationships that could be construed as a potential conflict of interest.
