# Supplementary material for: Antimicrobial Peptide LL-37 Facilitates Intracellular Uptake of RNA Aptamer Apt 21-2 Without Inducing an Inflammatory or Interferon Response
Source: Front Immunol. 2019 Apr 24;10:857. doi: 10.3389/fimmu.2019.00857 (PMC6491520; doi:10.3389/fimmu.2019.00857)
Supplement: Supplementary file 1 [file Data_Sheet_1.PDF]

Figure S1: Gating strategy for pDCs

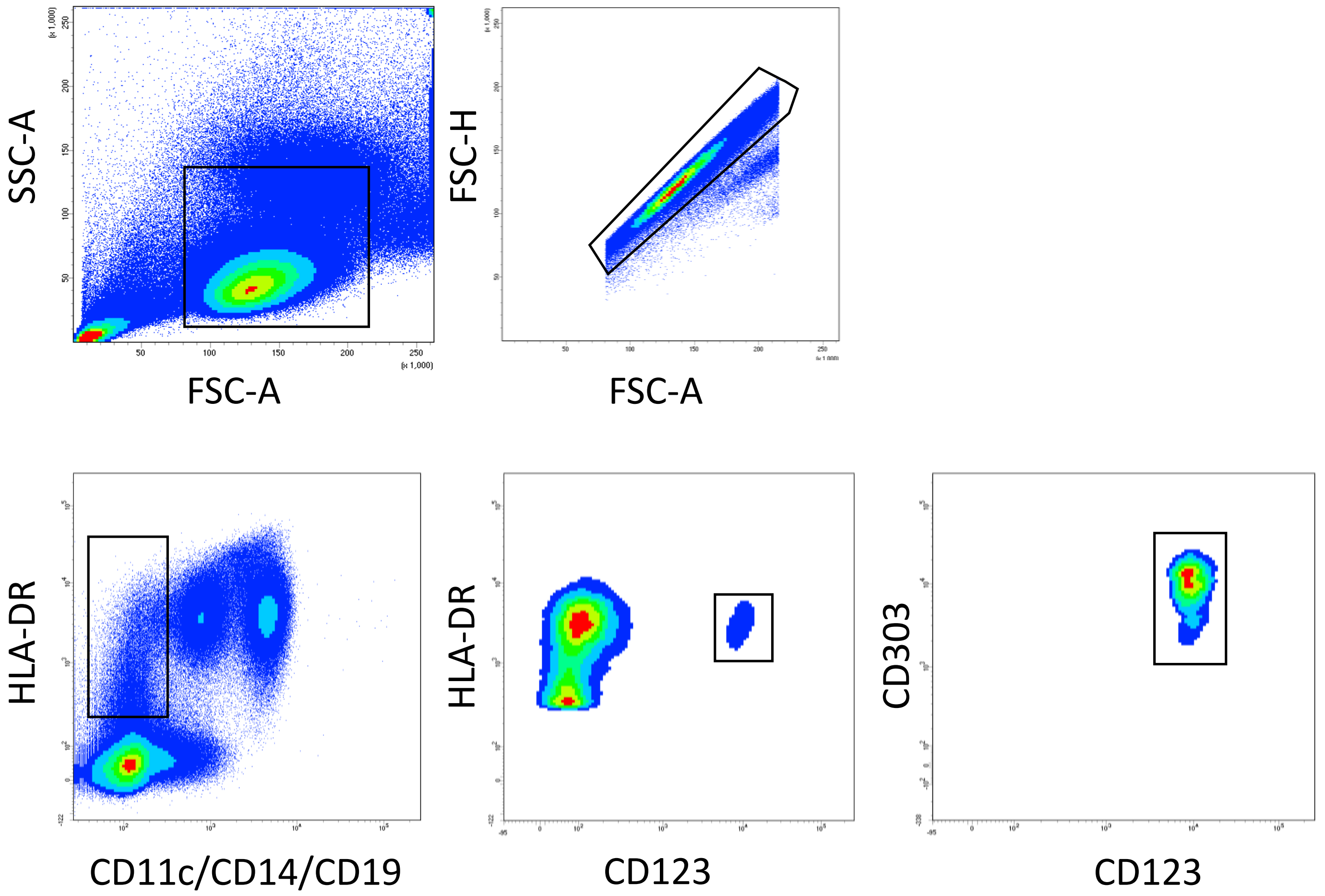

Figure S1: PBMCs were stained for surface markers HLA-DR, CD11c, CD14, CD19, CD303 and CD123. A simple gate was used to isolate lymphocytes and granulocytes, followed by FSC-H/FSC-A for doublet discrimination. HLA-DR^{high}, CD11c/CD14/CD19^{low} cells were then gated for exclusion of monocytes, classical DCs, and B cells, followed by HLA-DR^{high} CD123^{high}. Finally, CD303^{high} CD123^{high} cells were gated as pDC population.
